# Using an onset-anchored Bayesian hierarchical model to improve predictions for amyotrophic lateral sclerosis disease progression

**DOI:** 10.1186/s12874-018-0479-9

**Published:** 2018-02-06

**Authors:** Alex G. Karanevich, Jeffrey M. Statland, Byron J. Gajewski, Jianghua He

**Affiliations:** 1 Department of Biostatistics, University of Kansas Medical Center, Kansas City, KS USA; 2 Department of Neurology, University of Kansas Medical Center, Kansas City, KS USA

**Keywords:** ALS, Hierarchical modelling, ALSFRS, Prediction, Pro-act, Onset-anchor

## Abstract

**Background:**

Amyotrophic Lateral Sclerosis (ALS), also known as Lou Gehrig’s disease, is a rare disease with extreme between-subject variability, especially with respect to rate of disease progression. This makes modelling a subject’s disease progression, which is measured by the ALS Functional Rating Scale (ALSFRS), very difficult. Consider the problem of predicting a subject’s ALSFRS score at 9 or 12 months after a given time-point.

**Methods:**

We obtained ALS subject data from the Pooled Resource Open-Access ALS Clinical Trials Database, a collection of data from various ALS clinical trials. Due to the typical linearity of the ALSFRS, we consider several Bayesian hierarchical linear models. These include a mixture model (to account for the two potential classes of “fast” and “slow” ALS progressors) as well as an onset-anchored model, in which an additional artificial data-point, using time of disease onset, is utilized to improve predictive performance.

**Results:**

The onset-anchored model had a drastically reduced posterior predictive mean-square-error distributions, when compared to the Bayesian hierarchical linear model or the mixture model under a cross-validation approach. No covariates, other than time of disease onset, consistently improved predictive performance in either the Bayesian hierarchical linear model or the onset-anchored model.

**Conclusions:**

Augmenting patient data with an additional artificial data-point, or onset anchor, can drastically improve predictive modelling in ALS by reducing the variability of estimated parameters at the cost of a slight increase in bias. This onset-anchored model is extremely useful if predictions are desired directly after a single baseline measure (such as at the first day of a clinical trial), a feat that would be very difficult without the onset-anchor. This approach could be useful in modelling other diseases that have bounded progression scales (e.g. Parkinson’s disease, Huntington’s disease, or inclusion-body myositis). It is our hope that this model can be used by clinicians and statisticians to improve the efficacy of clinical trials and aid in finding treatments for ALS.

**Electronic supplementary material:**

The online version of this article (10.1186/s12874-018-0479-9) contains supplementary material, which is available to authorized users.

## Background

Amyotrophic Lateral Sclerosis (ALS), is a rare neuro-degenerative disease which exhibits extreme between-subject variability. Progression of ALS is typically measured by the ALS Functional Rating Scale (known as the ALSFRS, or with additional respiratory questions, the revised ALSFRS-R). The ALSFRS is a physician-reported outcome on a scale of 0 – 40 which grades common activities of daily living like dressing, eating, and walking. An ALSFRS score of 40 corresponds to normal function, and this score will decrease as the disease progresses. The ALSFRS, which is usually non-increasing, has been shown to decrease in a linear fashion over the course of a typical clinical trial (6 months to 1 year) [1, 2], although the linearity is disputed over long periods of time [3].

Faster disease progression is consistently associated with lowered survival [2, 4–8], although many of the clinical measurements shown to be associated with survival (e.g. region of symptom onset and Riluzole use. Riluzole is the only FDA-approved drug for ALS) are not significantly associated with disease progression [9–11]. As rates of progression on the ALSFRS are often used in phase II and III clinical trials, more accurate predictive models would help researchers in improving trial efficiency. For purposes of imputation and adaptive trial simulation, it may be more desirable to consider prediction of the actual ALSFRS as an endpoint, rather than its slope. Furthermore, ALS patients and their doctors may also gain more utility out of predicting individual ALSFRS scores rather than slope.

Our aim was to develop a predictive Bayesian hierarchical model which could be used to predict individual ALSFRS scores after 1 year from trial beginning using at most the first 3 months of clinical trial data. Our baseline model is a Bayesian hierarchical linear model, which is similar to a linear mixed effects model. We then compared the predictive power of this baseline model to those provided by a Bayesian mixture model and a Bayesian onset-anchored hierarchical linear model. The onset-anchored model leverages an additional data-point for each patient which assumes maximum ALSFRS score at the time of disease onset. Note that the approach of using an onset-anchor is applicable in modelling other diseases which utilize a bounded rating scale (Parkinson’s disease, Huntington’s disease, etc.). We additionally consider variable selection to improve model predictive accuracy, as well as consider model robustness when less than 3 months of data are available.

## Methods

### Study population

The datasets analyzed during this study are available in the Pooled Resource Open-Access ALS Clinical Trials database (PRO-ACT) (https://nctu.partners.org/ProACT/) [12]. In 2011, Prize4Life, in collaboration with the Northeast ALS Consortium, and with funding from the ALS Therapy Alliance, formed the PRO-ACT Consortium. The data available in the PRO-ACT Database has been volunteered by PRO-ACT Consortium members. As of December 2015, PRO-ACT had 4838 unique subjects, each having at least one reported ALSFRS or ALSFRS-R score. As PRO-ACT is a collection of data from clinical trials, we further subset this data to only include subjects that were receiving placebos. This resulted in 1301 subjects to be considered for analysis. One patient was later dropped due to having no data entered for self-reported disease onset time, bringing the final number of subjects to 1300. For more demographic information on these subjects, see Table 1.

**Table 1:** Demographic data for *n*= 1300 ALS subjects from the PRO-ACT database considered for analysis

Categorical Counts			
Sex	Male: 812 (63%)	Female: 488 (37%)	
Race	White: 1218 (94%)	Black: 22 (2%)	Latino: 13 (1%)
	Asian: 12 (1%)	Indian: 1 (< 1%)	Other: 34 (3%)
Riluzole Use	Yes: 600 (46%)	No: 358 (28%)	Not Reported: 342 (26%)
Continuous	Mean	SD	
Age (at trial start)	55.5	11.9	
Self-Reported Disease Onset Time (days from trial start)	− 658.4	456	
Number of ALSFRS scores	9.3	4.5	

For these 1300 subjects, we used ALSFRS scores to measure disease progression. The ALSFRS score is bounded between 0 and 40, and is typically non-increasing. Patients with ALSFRS-R scores, the revised ALSFRS, had their scores converted to the ALSFRS by summing the scores from the first nine questions of the ALSFRS-R (which concern motor and bulbar function) as well as the score from the first respiratory question, R1: Dyspnea.

### Model comparison

Our objective was to build a predictive model with which we could use the first 3 months of a subject’s data to determine their ALSFRS score at 1 year. As very few subjects had a measurement at exactly 1 year, we instead used the model to predict each subject’s first score after day 365, denoted as *FRS*_365_. We chose to predict after 12 months because that is a commonly used endpoint in ALS trials (specifically, only 4 of 18 recent ALS trials had endpoints shorter than 1 year [13]; due to the linearity of the ALSFRS decline over timespans shorter than 1 year it stands to reason that a linear model which performs well at 12 months would perform well for shorter endpoints). Three months was chosen as the cutoff because: 1) this was the window used in the DREAM ALS Stratification Prize4Life Challenge [14]; 2) 3 months represented a reasonable amount of time for making 12 month predictions; and 3) is a time frame with utility for both adaptive trial designs and for imputing missing data. Ideally, this model would be accurate even when less than 3 months of subject data are available.

Large amounts of variability are inherently associated with any ALS model. Bayesian hierarchical models excel at capturing many sources of variability, which can then be reported via posterior predictive credible intervals. A credible interval is preferable for its interpretability: in the framework of a Bayesian model, there is a 95% chance that a subject’s *FRS*_365_ is within the 95% credible interval. Note that while the gold standard for confidence intervals is 90% or 95%, this is done to control the type I error rate. A credible interval, being a statement of probability, has no such restriction, and thus is useful with even lower credible levels, such as 70% or 80% [15].

We considered three types of models, which are described below: A Bayesian hierarchical linear model, a Bayesian hierarchical mixture model, and a Bayesian onset-anchored hierarchical linear model. Note that these models are all linear with respect to time. This is largely because a patient in PRO-ACT typically has only one ALSFRS measurement per month, which causes more complicated models, such as 3-parameter sigmoidal curves, to suffer from convergence problems. Linearity is also convenient because the slope parameter can be used as a simple-to-interpret measure of the disease’s rate of progression.

The models were compared by the distribution of their posterior mean-square-error (MSE) resulting from a cross-validation analysis. Cross-validation entails splitting the data in to a training set with which to build the model, and a validation set with which to assess the model’s predictive power [15]. We looked at 10 randomly-sampled validation sets for each model, and found the results off the cross-validation to be very robust across the various training/validation splits. The posterior distribution of the MSE, denoted $\overset{\sim }{MSE}$, is defined as follows: for each subject *i*, take the square of the difference between their true *FRS*_365, *i*_ and their posterior predictive distribution for *FRS*_365, *i*_, denoted ${\overset{\sim }{FRS}}_{365,i}$. Sum this over all subjects in the validation set, adjusting for the size of the validation set. In other words $\overset{\sim }{MSE}=\sum \limits_{i=1}^n\frac{{\left({\overset{\sim }{FRS}}_{365,i}- FR{S}_{365,i}\right)}^2}{n}$.

In order to be in the validation set, subjects needed at least one ALSFRS score after 1 year from baseline. Again, as the ALSFRS score at 1 year was not specifically observed for most patients, we instead predicted *FRS*_365_, the subject’s first score after 365 days. Of subjects who had at least 1 year of data, average *FRS*_365_ was 386.7 days, with standard deviation of 23.7 days and maximum of 577 days. The same training and validation sets were used to validate all three models.

All analysis was done using R [16], OpenBUGS [17], and the R package R2openBUGS [18]. Pseudo-code which describes the model in more detail is provided in the Additional file 1.

#### Bayesian hierarchical linear model

Since ALS seems to progress linearly over most of the 1 year time frames in the PRO-ACT database, we started with a linear hierarchical Bayes mixed effects model with weak and uninformative priors. Specifically, the ALSFRS for subject *i* at time *t* is modeled as

$$ ALSFR{S}_i(t)\sim {T}_3\left({a}_i+{b}_it,{\sigma}^2\right) $$

**Fig. 1:**
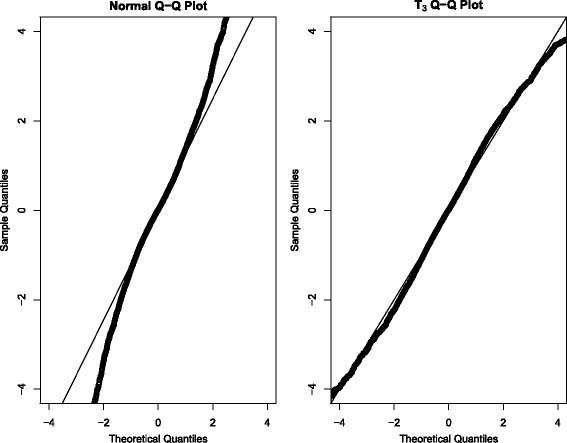
QQ plots for the residuals obtained from fitting simple linear regression models on subjects that had at least 5 ALSFRS measurements

Truncated to *ALSFRS*_*i*_(*t*) ∈ [0, 40], which is easily done in OpenBUGS. *T*_3_ denotes the centered non-standardized t-distribution with 3 degrees of freedom and non-standardized variance *σ*^2^. Note that a standardized t-distribution with 3 degrees of freedom would instead have a variance of 1. Parameters *a*_*i*_ and *b*_*i*_ are the subject-specific intercept and slope term. A t-distribution with 3 degrees of freedom was chosen because a normal distribution was too narrow in the tails. Additionally, we observed that the residuals from simple linear regression on subjects (with sufficient amounts of data) followed a *T*_3_ distribution extremely well (see Fig. 1). To further justify this, we also observed a massive decrease in model deviance information criteria (DIC) when using *T*_3_ versus the normal distribution. A reduction of more than 10 to DIC is typically associated with improved model fit; using the *T*_3_ over the normal resulted in a DIC reduction of over 1700.

Continuing the model description, the hyperparameters *a*_*i*_ and *b*_*i*_, in turn, have the following distributions:

$$ {a}_i\sim N\left({p}_0,{\sigma}_0^2\right) $$

$$ {b}_i\sim N\left({p}_1,{\sigma}_1^2\right) $$

Where *a*_*i*_ is truncated to *a*_*i*_ ∈ [0, 40] and *b*_*i*_ is truncated to *b*_*i*_ ∈ (−∞, 0]. Weak priors from the literature and discussions with clinicians were assumed for *p*_0_ and *p*_1_. Specifically Castrillo-Viguera et al. [19] reported that the ALSFRS-R decline in one database is roughly − 0.92 units per month with standard error of 0.08. This translates to roughly an ALSFRS decline of −.025 per day, and leads us to the following priors:

$$ {p}_0\sim N\left(33,{3}^2\right) $$

$$ \kern1em {p}_1\sim N\left(-0.025,{0.3}^2\right) $$

Where the increased error in *p*_1_ allows for more strength in the analysis to come from the data. Generally subjects with low baseline ALSFRS scores are not enrolled in clinical trials, and the prior for *p*_0_ was chosen to reflect this while still allowing a wide range of potential starting ALSFRS values. Uninformative priors were assigned to the remaining variables: ${\sigma}^2,{\sigma}_0^2,$ and ${\sigma}_1^2$ are each given the prior Γ^−1^(0.001,1000), which is equivalent to $\frac{1}{\sigma^2}\sim \Gamma \left(\mathrm{0.001,0.001}\right)$.

Such a Bayesian model, aside from the weakly informed priors on *p*_*i*_, was suggested by Gomeni et al. [20] . A key advantage to hierarchical modelling in this way is that it allows for shrinkage of error resulting from sample means [21, 22], and also lets subjects with fewer data points “borrow” information from the remaining population. The Bayesian analysis also has advantages with respect to interpretability (especially in a clinical setting). This model will be referred to as the “linear model”.

#### Bayesian hierarchical linear mixture model

A mixture model is useful when each subject belongs to one of several groups, each group having their own specific progression distributions. Specifically, Gomeni et al. [20], suggested that ALS subjects could be classified as either “fast” or “slow” progressors. To model this, we assume each subject is either a fast or slow progressor, and assume that each group has their own average rate of disease progression (parameterized by the mean of the subject-specific slope). We further assume the slope parameter for fast progressors is strictly steeper (more negative) than those of slow progressors.

The ALSFRS for subject *i* at time *t* is still *ALSFRS*_*i*_(*t*)~*T*_3_(*a*_*i*_ + *b*_*i*_*t*, *σ*^2^) truncated to *ALSFRS*_*i*_(*t*) ∈ [0, 40], but now we let ${b}_i\sim N\left(\Lambda, \kern0.5em {\sigma}_1^2\right)$ truncated to *b*_*i*_ ∈ (−∞, 0]. This starts the mixture process, with Λ being either Λ_1_ or Λ_2_ = (Λ_1_ + *c*), where *c* is a positive constant, with probability Pr(Λ = Λ_*i*_) = *π*_*i*_. Finally, we use the following priors: *π*_*i*_~*Dirichlet*(1, 1), ${\Lambda}_1\sim N\left(0,{\sigma}_{\Lambda_1}^2\right)$. The error terms ${\sigma}^2,{\sigma}_1^2,{\sigma}_0^2,{\sigma}_{\Lambda_1}^2$ are all assigned uninformative priors of Γ^−1^(0.001,1000) and we assign *c*~*N*(0,100) truncated to *c* ∈ [0, ∞) . All other priors and parameters are specified as in the linear model (2.2.1). This model shall be referred to as the “mixture model”.

#### Bayesian onset-anchored hierarchical linear model

**Fig. 2:**
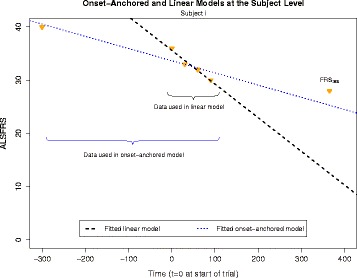
Ordinary least-squares estimates for two models: the linear model uses data from zero to 3 months only, while the onset-anchored model includes an additional artificial data-point. This time point is given as (*x*, *y*)= (time-point of disease onset-time, maximal ALSFRS of 40)

This model resembles the linear model in structure, but uses an idea first introduced by Proudfoot et al. [23]. The idea was to create an additional *artificial* data-point, referred to as the “onset-anchor”. We do this by assuming that each subject had an ALSFRS score of 40 (the maximum possible score) at their time of disease onset (see Fig. 2). Aside from this artificial data point, the parameters and model specification remain identical to those given in the linear model. This model is referred to as the “onset-anchored model”.

Assuming the maximum possible ALSFRS score at disease onset time was an idea first introduced by Proudfoot et al. [23]. They used this assumption to create a slope between the onset anchor and the first observed ALSFRS score, which was then used as a predictor for measuring a patient’s disease progression. Our onset-anchored model, however, treats this additional artificial data-point as an observed value (specifically, a leverage point) in the modelling framework.

Considering the simplicity of this approach, the addition of a non-random leverage point to aid in model prediction is a surprisingly novel technique. This method will, however, result in a biased linear regression model: specifically we would not expect that the difference between the observed *FRS*_365_ and mean of the posterior predictive distribution of *FRS*_365_ to be zero on average (in other words $E\left({\overset{\sim }{FRS}}_{365,i}- FR{S}_{365,i}\right)$ is not necessarily zero). Recall that the SE of any prediction is composed of the sum of the square of the prediction bias and the prediction variance. In order for this biased model to predict *FRS*_365_ well, the reduction in prediction variance needs to dramatically outweigh the increase in prediction bias.

### Covariate selection using the onset-anchored model

After choosing a “winner” from the three models mentioned above (the onset-anchored model), we wished to determine which clinical features, if any, improved predictive accuracy when used as covariates in the model. Clinical features considered were height, symptom onset time, sex, age, race, individual sub-questions of the ALSFRS, forced vital capacity (FVC, both liters and percent predicted of normal), respiratory rate, weight, Riluzole use (yes/ no), and site of onset (bulbar/ limb). Many lab measurements are included in PRO-ACT, yet due to their sparse nature, only lab features which were present in at least 90% of the subjects were considered. Albumin has been shown to be associated with ALS survival [24] and was included for analysis even though it was only present in 86% of subjects. The following lab features were considered in our analysis: chloride, serum aspartate aminotransferase (AST), glucose, sodium, blood urea nitrogen, potassium, bilirubin, alanine transaminase (ALT), creatinine, and albumin.

Many of these features were repeated measures. To use them as covariates, they were truncated to at most 3 months (for both the training and the testing set) and then collapsed to slope and intercept (baseline) measures. Specifically, we performed a linear regression on the feature with respect to time (truncated at 3 months), and extracted the ordinary least squares estimates for the slope and intercept. While true baseline data would be preferable over the ordinary least squares intercept estimator, baseline data was frequently not available. Therefore the ordinary least squares intercept estimator was chosen for homogeneity. Collapsing longitudinal predictors has been successfully employed in other ALS predictive models [25, 26], and greatly simplifies the modeling process. All features were normalized using their sample means and variances for ease of analysis and interpretability.

As we were more interested in predictive power, our criteria for feature selection was improvement to the average MSE resulting from predicting *FRS*_365_ in 100 replicates of cross validation using repeated random sub samples (Monte Carlo cross-validation). This method was chosen rather than choosing covariates based on statistical significance as given by a small *p*-value. Deviance information criterion (DIC) was also considered in assessing whether features improved the model or not.

The specifics of our covariate random sub-sampling cross validations are as follows: For the covariate of interest, a single replicate (of 100) first begins by randomly subsetting the overall data in to 300 subjects with non-missing entries. While multiple imputation could be used here, we chose to only use complete cases to drastically reduce computation time as well as eliminate potential convergence problems. Of this subset, we randomly chose a validation and testing set (30 subjects in validation, 270 in testing), built onset-anchored models both using and not using the covariate, and compared the average difference in posterior MSE. This is a single replicate, and we repeat this 100 times for each covariate. We then analyzed the average effect of including the covariate over these 100 replicates (for each covariate). Specifically, we considered the two onset-anchored models (for the full model specification, see Additional file 1):

Covariate onset-anchored model:

$$ ALSFR{S}_i(t)\sim {T}_3\left({b}_{0i}+{b}_{1i}t,{\sigma}^2\right);{ALSFRS}_i\in \left[0,40\right] $$

$$ \kern4.25em {b}_{0i}\sim N\left({p}_{00}+{p}_{01}{X}_i,{\sigma}_0^2\right);{b}_{0i}\in \left[0,40\right] $$

$$ \kern4.25em {b}_{1i}\sim N\left({p}_{10}+{p}_{11}{X}_i,{\sigma}_1^2\right);{b}_{1i}\in \left(-\infty, 0\right] $$

Baseline (no covariate) onset-anchored model:

$$ ALSFR{S}_i(t)\sim {T}_3\left({b}_{0i}+{b}_{1i}t,{\sigma}^2\right);{ALSFRS}_i\in \left[0,40\right] $$

$$ \kern4.25em {b}_{0i}\sim N\left({p}_{00},{\sigma}_0^2\right);{b}_{0i}\in \left[0,40\right] $$

$$ \kern4.25em {b}_{1i}\sim N\left({p}_{10},{\sigma}_1^2\right);{b}_{1i}\in \left(-\infty, 0\right] $$

Where *X*_*i*_ is the subject-specific covariate, *t*_*i*_ is time for subject *i*. The slope of subject *i* is *b*_1*i*_ which, in the covariate model, is a function depending on *X*_*i*_. Similarly, *b*_0*i*_ is the subject-specific intercept. As per hierarchical modelling, we assume priors only for the hyperparameters *p*_*jk*_ (*j* = 0, 1 and *k* = 0, 1). As per the linear model, the following weak priors were assumed:

$$ {p}_{00}\sim N\left(33,{3}^2\right) $$

$$ {p}_{10}\sim N\left(-0.025,{0.3}^2\right) $$

Uninformative priors were assigned for the remaining parameters in both models: *σ*^2^ and ${\sigma}_i^2$ are given Γ^−1^(0.001,1000); *p*_01_ and *p*_11_ are given *N*(0,100^2^) (see Additional file 1).

## Results

**Fig. 3:**
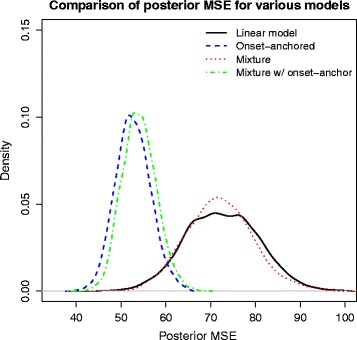
Comparison of posterior MSE distribution for four types of hierarchical models: linear, onset-anchored, mixture, and mixture with the additional data-point used in the onset-anchored model. This is from a single replicate of the cross-validation analysis, but this separation of distributions is typical

We investigated the predictive power of three types of Bayesian hierarchical models: linear, mixture, and onset-anchored. In a Bayesian framework, when cross-validating a model, the resultant MSE has a posterior distribution which takes in to account all of the sources of variation within the model. Specifically, these sources of variation include 1) variation within the model; 2) variation of the posterior parameters; and 3) the variation of the posterior predictive distribution. Therefore it is important to not only lower the MSE but to also decrease its variance. Of the three models, the onset-anchored model not only had the smallest MSE but also had the MSE with the smallest variance (Fig. 3). Note that the DIC between the onset-anchored model and the standard linear model cannot be compared, because the additional data-point in the onset-anchored model results in a different likelihood.

The MSE for the onset-anchored model is not only smaller in terms of expectation (In Fig. 3 the means of the MSE for the onset-anchored, mixture model, and linear model were 51.1, 68.5, and 73.7 respectively) but also has the smallest variance. We also considered a mixture model which utilized the additional data-point given by the onset-anchor. This complex model performed about as well as the more parsimonious onset-anchored model, which can be seen by their nearly overlapping MSE distributions in Fig. 3.

Since from Fig. 1 shows that the onset-anchored model to be the winner, we decided to check the robustness of this result by repeated random sub-sampling cross validation (25 replications). We specifically compared the posterior mean MSE between the linear and onset-anchored models for randomly selected training/testing splits. On average, the onset-anchored model had a posterior mean MSE that was 20.7 (standard deviation 4.1) less than the linear model (a figure is provided in the Additional file 1).

We next attempted to find which covariates or features could consistently improve the MSE of the onset-anchored model, or decrease the DIC. While many clinical and lab predictors had nonzero effects on the posterior slope and intercept (meaning *p*_11_ and/ or *p*_01_ were nonzero), very few predictors consistently improved the MSE, and among those that did, the improvement to the MSE was very small (Table 2). Some variables, such as FVC: Subject Liters (slope) and FVC: Percent Normal (slope) reduced DIC (each reduced DIC by about 3.45), however they did not contribute towards a meaningful improvement in predictive power. Of the 53 covariates tested, only “disease onset time” resulted in an improvement to the MSE which was on average greater than 1%. This is most likely due to disease onset time giving a slight bias correction to the model. The next best covariates were subject’s 3-month slope of FVC (in raw liters) and 3-month slope of the first question from the ALSFRS: Q1, Speech.

**Table 2:** Median reduction to MSE, in percentage, for covariates which improved the MSE in the onset-anchored model. The inter-quartile range (IQR) for the percent reduction as well as average difference in DIC is shown as well

Covariate Name	Median % MSE reduction (negative values signify an increase to the MSE)	IQR for % MSE reduction	Mean DIC adjustment (larger values result in larger DIC)
Onset Time	0.0174	0.027	2.8
FVC: Subject Liters (slope)	0.0095	0.02	−3.5
Q1: Speech (slope)	0.0081	0.0187	0.8
Diagnosis Time	0.0059	0.0183	−2.1
Q7: Turning in Bed(slope)	0.0052	0.0182	−3.5
Q8: Walking (slope)	0.0052	0.0254	−2.2
AST (slope)	0.0043	0.0179	−0.3
Q5: Cutting (slope)	0.0043	0.0202	−1.5
Q6: Dressing/Hygiene (slope)	0.0039	0.0223	−1.9
ALT (slope)	0.0034	0.0166	0.1
Q2: Salivation (slope)	0.0032	0.0195	1.7
Q9: Climbing Stairs (slope)	0.003	0.0232	−2
AST (intercept)	0.0028	0.0162	0.8
FVC: Percent Normal (slope)	0.0025	0.0225	−3.4
Race	0.0021	0.0156	−0.1
ALT (intercept)	0.0021	0.0182	−0.7
Bilirubin Total (slope)	0.0019	0.0196	−0.5
Respiratory Rate (intercept)	0.0017	0.0146	0.3
Q2: Salivation (intercept)	0.0013	0.0203	−1.4
Creatinine (intercept)	0.0011	0.0152	−0.7
Age	0.001	0.0185	−2.1
Q1: Speech (intercept)	0.001	0.0224	2.2
Potassium (slope)	0.001	0.0136	−0.9
Onset Site: Bulbar	0.001	0.0171	−0.5
Height	0.0009	0.0169	0.8
Weight (slope)	0.0009	0.0174	−1.7
Sodium (intercept)	0.0008	0.0188	1
Bilirubin Total (intercept)	0.0008	0.0183	0.8
Sex	0.0006	0.0169	−0.4
Q10: Respiratory (slope)	0.0006	0.0153	0.8
Q4: Handwriting (slope)	0.0006	0.0206	−0.7
Weight (intercept)	0.0006	0.0181	−0.5
Q5: Cutting (intercept)	0.0002	0.0224	−4.1

Recall that several of these clinical values have been found to be associated with survival, including Forced Vital Capacity (FVC), age of onset, and site of onset (bulbar or limb, which can help differentiate subtypes of ALS). However, none of these covariates have been consistently useful for modelling ALSFRS progression [9], and this is consistent with our findings. Riluzole use, in particular, worsened MSE by a median of 0.09% (see Additional file 1 for expanded Table 2). Again, this is not surprising as Riluzole has only a weak effect on survival and has not been shown to be consistently associated with decreased disease progression [10, 27].

**Fig. 4:**
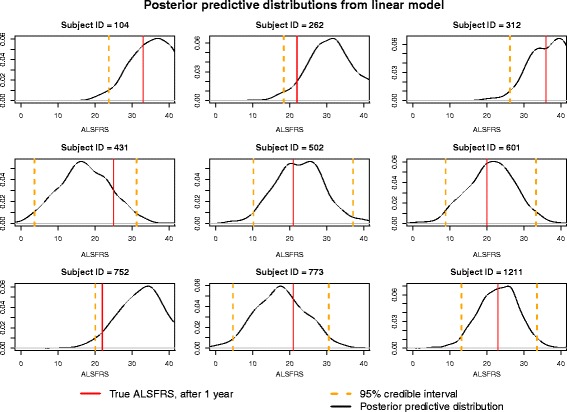
Posterior predictive distributions for a random sample of subjects’ *FRS*_365_ obtained through cross-validation utilizing the standard linear model

**Fig. 5:**
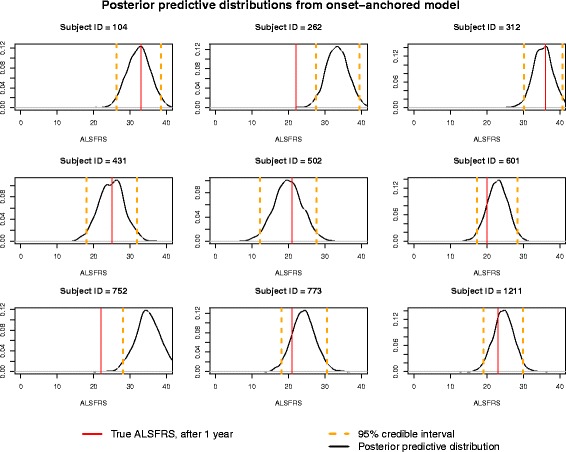
Posterior predictive distributions for a random sample of subjects’ *FRS*_365_ obtained through cross-validation utilizing the onset-anchored model

To appropriately predict the ALSFRS for a given subject after 1 year from trial onset using data collected up to 3 months after trial onset, a measure of uncertainty must be reported as well. Since a Bayesian analysis instead was performed, we can obtain 95% credible intervals for each subject’s predicted *FRS*_365_ (equivalently the posterior predictive interval for ${\overset{\sim }{FRS}}_{365}$). Figs. 4 and 5 give a sample of posterior distributions from a cross-validation for nine randomly-selected subjects’ ${\overset{\sim }{FRS}}_{365}$, as well as their 95% credible intervals and true *FRS*_365_ (the subject’s first score at, or after, 365 days). To further demonstrate the improved predictive power of the onset-anchored model, this is done for both the standard linear model (Fig. 4) as well was the onset-anchored model (Fig. 5). It can be noted that the credible intervals for the linear model are very wide, encompassing nearly the full range of the disease. As the time of data collection used to make the prediction increases from 3 months, this prediction becomes more accurate.

**Fig. 6:**
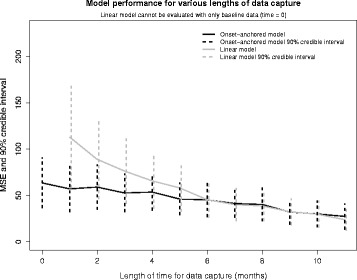
The effect of changing the number of months of data used for prediction in both the linear and onset-anchored models. This effect is measured by the MSE (and associated 90% credible interval) resulting from a single replicate of the cross-validation analysis for both models. As the length of data-capture increases the benefits from including an anchor decreases

The performance of the onset-anchored model is vastly superior to that of the linear model when the length of time for data collection is short. Figure 6 shows that the onset-anchored model, using only baseline data, typically outperforms a linear model using many months of subject data. Figure 6 also shows that the onset-anchored model performs well even when the window for data capture is restricted less than 3 months, including when only a baseline measurement is available for each subject. Finally, it also shows that as the more data is used to build the prediction, the benefit of including an anchor decreases. These models do not include any longitudinal covariates that were tested previously, so we are not relying upon measurements that do not exist yet.

Recall that, while MSE of prediction is drastically reduced when using the onset-anchored model, it is in fact a biased model. The additional data-point causes the model to typically underestimate the rate of disease progression, resulting in a higher predicted *FRS*_365_ than observed. Using the onset-anchored model resulted in a prediction bias of, on average, about 2 (on the ALSFRS scale). For comparison, the linear model was typically unbiased.

**Fig. 7:**
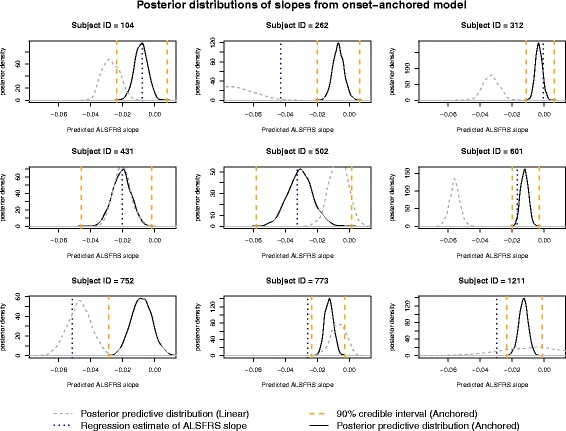
Posterior predictive distributions for a random sample of subjects’ ALSFRS slope obtained through cross-validation utilizing the onset-anchored model. The regression estimate of the ALSFRS slope (vertical dotted line) was calculated using ordinary least squares on subject’s full data

Finally, one way to measure progression of ALS is by the slope of the ALSFRS. An advantage to using the Bayesian hierarchical framework is that the ALSFRS slope for subject *i*, defined previously as *b*_1*i*_, is specified in the model likelihood and therefore has a posterior distribution. Thus, one can then obtain a posterior estimate and credible interval for subject *i*^′^ s slope from this distribution. In other words, when using this model one can easily predict slope for a given subject in addition to *FRS*_365_. Examples of the posterior predictive distributions for the ALSFRS slope using the onset-anchored model and 3 months of data, with 90% credible intervals, is provided in Fig. 7 for the same nine subjects used in Figs. 4 and 5. Also included in Fig. 7 are the posterior predictive slopes from the linear model. It should be noted that, on average, the MSE of predicting slope is smaller when using the onset-anchored model versus the linear model (using 3 months of data to predict slope at 1 year). As the onset-anchored model performs well even when using only baseline data, subject slopes could be predicted using this model as soon as a baseline ALSFRS score has been established.

## Discussion

We explored three different Bayesian hierarchical predictive models with the goal of modelling ALS disease progression. These models were linear, mixture, and onset-anchored. The onset-anchored model, which uses an additional data-point by assuming the maximum ALSFRS score at time of disease onset (e.g. 40), is the best model in terms of predictive accuracy via cross-validation. This is especially noticeable when the window for data capture is very small, such as only using a baseline ALSFRS score.

While linear over the course of a typical clinical trial, progression of the ALSFRS could become curvilinear over long periods of time. This is further reinforced by the fact that it is bounded between 0 and 40, and is typically non-increasing. Predictive models that attempt to account for this non-linear progression suffer from a disparity between the number of subject-specific data-points and the necessary number of model parameters. We hypothesize that using the onset-anchor helps to “balance” this prediction (see Fig. 2), while also enabling shrinkage on the slope estimator. The result is a model that has reduced variability of parameter estimates (at the cost of a small increase in bias), which enables a large reduction in overall prediction MSE.

Using 3 months of subjects’ data, we found that very few clinical features improved prediction as measured by the MSE of repeated cross-validation analysis. Among those features that did consistently improve the MSE, the improvement was rarely more than a 1% reduction. This corroborates findings by Creemers et al. [9] who found the quality of evidence among disease progression prognostic factors to be low at best. The covariate which offered the largest and most consistent improvement to the model’s prediction was disease onset time. As disease onset time is also a key part of the onset-anchor model, this stresses its importance, supporting other studies which have shown that onset time is strongly associated with disease progression as well as survival [5, 6, 25].

We found that the onset-anchored model performs well when predicting ALSFRS scores after 1 year even when less than 3 months of data is available. This is still true when the prediction is to be made for 6 months rather than 1 year; thus ALS trials which have endpoints earlier than 1 year can still benefit from the onset-anchored model. The predictive performance for 6 month prediction was proportional to that given in Fig. 6; when less than 2 months of data is available the onset-anchor model outperforms the linear model.

From a practical point of view, a model which only requires time of disease onset and at most 3 months of progression data eases both patient and clinician burden by requiring less overall measurements. The Bayesian modeling approach proposed here can help inform the design of adaptive studies, and be used as an imputation scheme to conduct trials more quickly [28–30]. Finally patients with ALS are routinely interested in charting their own progression, as well as trying interventions which might include treatments for spasticity or pain, or supplements geared towards slowing disease progression. In conjunction with a self-administered ALSFRS, the onset-anchored model then becomes a predictive tool that an ALS patient can use aid them in tracking their disease and assess the utility of self-administered interventions.

While the idea of using an additional data point as used by the onset-anchored model is simple, it is surprisingly novel. Assuming minimal disease progression at disease onset time (utilizing the upper bound of the ALSFRS) is a sort of intelligent imputation, but differs from traditional imputation in that we are not filling in missing data “gaps”. This is because none of the patients actually have an observed ALSFRS score at disease onset time.

Creating biased models to improve predictive MSE is not uncommon, and is used in ideas like fixed-point regression or ridge regression. However, using an artificially created data-point and treating it as observed data is something that, to the best of our knowledge, is something that has never been used before. We have found no literature where it is theoretically discussed or practically used. This methodology could be applied to any longitudinal data where the onset time of the process being modelled is known. Other diseases which have bounded rating scales which measure progression, including Parkinson’s disease or Huntington’s disease, might benefit tremendously from predictions that utilize an onset-anchor.

One limitation to the current study is that subjects who died before the clinical trial had progressed a full year were not candidates for cross validation, and hence did not directly contribute to the MSE. However, the Bayesian framework allows these subjects to be included in building the model, where their often increased rates of disease progression contribute to the variability of the model. Specifically, subjects who died prior to 1 year still contributed towards key model variables, including the distributions of rate of progression, effects of covariates, and variability measures throughout the model. Subjects who died prior to 1 year also had, on average, a lower predicted *FRS*_365_ than subjects who survived past 1 year. This is expected since a faster progression is associated with lowered survival.

Another limitation is the width of the posterior predictive distributions among individual subjects’ *FRS*_365_. These distributions express a combination of variation within the model, variation of the posterior parameters, and variation of the posterior predictive distribution. Due to the heterogeneity of ALS, it is not unexpected that *FRS*_365_ can range widely at the individual-patient level. This will remain a limitation of any predictive model until better factors which are more strongly associated with disease progression (rather than survival) are discovered.

The onset-anchored model’s inherent bias is another limitation of the model. This is the typical concern with any biased linear model, but in this case we can see that the reduction in the onset-anchored model’s MSE is worth the tradeoff. Problems associated with the bias include the interpretation of the 95% posterior predictive intervals of *FRS*_365_, (which are correct 73% of the time), as well as the underestimation of slope parameters. A possible solution might be to investigate a bias-correction term which would utilize disease-onset time as well as the number of days after the start of the trial that is associated with *FRS*_365_ (such as including an overall error term to disease onset time).

One final limitation worth pointing out is that disease onset time, a critical feature of the onset-anchored model, is a problematic variable. This variable typically comes from patient memory, and as a result is subject to recall bias. Proudfoot et al. point out that while this bias exists, using patient-recalled onset time is still a useful predictor for disease progression [23], and this is corroborated by our model.

Continuing this last point, we attempted to incorporate the recall bias of disease onset time in the onset-anchored model by including a normally distributed random error term associated with onset time (with vague priors on the parameters). We compared the models with and without this random error (similar to our covariate analysis), and found that including this random error typically improved the MSE by 5.8% (with interquartile range of 4.2%). This improvement is especially impressive when one recalls that the “best” covariate (disease onset time) only improved the model MSE by 1.7%. However, we only observe this improvement when the error term is shared across all subjects; if each subject is given their own individual error term then the models perform exactly the same. This leads us to believe that this error term is serving as a bias-correction term to the model.

## Conclusions

In this paper we considered the problem of predicting an ALS patient’s ALSFRS score at 1 year, given up to 3 months of data. Three different Bayesian hierarchical predictive models were considered: linear, mixture, and onset-anchored. The onset-anchored model, which leverages an additional artificial data-point which assumes the maximum ALSFRS score of 40 at the patient’s time of disease onset, is the best model with respect to predictive accuracy under cross-validation. The onset-anchored model is simple to implement, and is potentially applicable to various other diseases which measure progression by bounded rating scales.

The effect of many covariates (lab values, demographic information, etc.) on these predictions was assessed via repeated cross-validation. The result is that time of disease onset is the only covariate which provides a consistent improvement to predictions, but this is a very small improvement. This highlights the urgent need to develop a better understanding of the mechanism behind ALS progression.

The onset-anchored model has an added benefit over the other models in that it allows predictions as early as directly after the baseline measure. In other words, as soon as the first ALSFRS measure is taken in a clinical trial, the model can be utilized for endpoint prediction of the ALSFRS. We hope this model can be used by clinicians and statisticians to improve the efficacy of clinical trials and aid in finding treatments for ALS.

## Additional file

Additional file 1: Full model descriptions, psuedocode, and full covariate table. (DOCX 18 kb)

## Abbreviations

ALS: amyotrophic lateral sclerosis
ALSFRS: ALS Functional Rating Scale
ALSFRS-R: ALS Functional Rating Scale – Revised
MSE: Mean squared error
PRO-ACT: Pooled Resource Open-Access ALS Clinical Trial Database
