# Supplementary material for: Using an onset-anchored Bayesian hierarchical model to improve predictions for amyotrophic lateral sclerosis disease progression
Source: BMC Med Res Methodol. 2018 Feb 6;18:19. doi: 10.1186/s12874-018-0479-9 (PMC5801819; doi:10.1186/s12874-018-0479-9)
Supplement: Additional file 1: — Full model descriptions, psuedocode, and full covariate table. (DOCX 18 kb) [file 12874_2018_479_MOESM1_ESM.docx]

**Additional file 1**

**Full model description**

Let $X_{i}$ be a covariate and $ALSFRS_{i}(t)$ the ALSFRS score for subject $i$ at time $t$. In this, $\sigma^{2}$ is the precision parameter of the non-standardized $T$ distribution (3 degrees of freedom), which is defined as the inverse of the variance. The linear and onset-anchored hierarchical models are then defined as follows:

$$ALSFRS_{i}\left( t \right)\sim T_{3}\left( b_{0i}+b_{1i}t,\sigma^{2} \right);ALSFRS_{i}\left( t \right)\in[0,40]$$

$$b_{0i}\sim N\left( p_{00}+p_{01}X_{i},\sigma_{0}^{2} \right);b_{0i}\in[0,40]$$

$$b_{1i}\sim N\left( p_{10}+p_{11}X_{i},\sigma_{1}^{2} \right);b_{1i}\in\left( -\infty,0 \right]$$

$$p_{00}\sim N\left( 33, 0.111 \right)$$

$$p_{01}\sim N\left( 0, 0.0001 \right)$$

$$p_{10}\sim N\left( -0.025, 11 \right)$$

$$p_{11}\sim N(0,0.0001)$$

$$\sigma^{2}\sim\Gamma\left( 0.001, 0.001 \right)$$

$$\sigma_{0}^{2}\sim\Gamma\left( 0.001, 0.001 \right)$$

$$\sigma_{1}^{2}\sim\Gamma(0.001, 0.001)$$

**BUGS code (R2 OpenBUGS format)**

for (i in 1:N) #N is number of subjects.

{

for (j in 1:J[i]) #J is a vector containing the number of entries for each subject.

{

ALSmu[Jtot[i]+j] <-b0[i]+b1[i]*delta[Jtot[i]+j]

ALS[Jtot[i]+j] ~ dt(ALSmu[Jtot[i]+j], err, 3)%_%I(0,40)

}

b1_mean[i] <- p10 + p11*covariate[Jtot[i]+1]

b0_mean[i] <- p00 + p01*covariate[Jtot[i]+1]

b1[i]~dnorm(b1_mean[i],err1) %_%I(,0)

b0[i]~dnorm(b0_mean[i],err0) %_%I(0,40)

#predictive draws

predmu[i] <-b0[i]+b1[i]*365

pred365[i] ~ dt(predmu[i], err, 3) %_%I(0,40)

}

##priors

p00 ~ dnorm(33, 0.111)

p01 ~ dnorm(0, 0.00001)

p10 ~ dnorm(-0.025, 11)

p11 ~ dnorm(0, 0.00001)

err~dgamma(0.001, 0.001)

err1~dgamma(0.001, 0.001)

err0~dgamma(0.001, 0.001)

**Full table of data**

| **Covariate Name** | **Median % MSE reduction (negative values signify an increase to the MSE)** | **IQR for % MSE reduction** | **Mean DIC adjustment (larger values result in larger DIC)** |
| --- | --- | --- | --- |
| Onset Time | 0.0174 | 0.027 | 2.8 |
| FVC: Subject Liters (slope) | 0.0095 | 0.02 | -3.5 |
| Q1: Speech (slope) | 0.0081 | 0.0187 | 0.8 |
| Diagnosis Time | 0.0059 | 0.0183 | -2.1 |
| Q7: Turning in Bed(slope) | 0.0052 | 0.0182 | -3.5 |
| Q8: Walking (slope) | 0.0052 | 0.0254 | -2.2 |
| AST (slope) | 0.0043 | 0.0179 | -0.3 |
| Q5: Cutting (slope) | 0.0043 | 0.0202 | -1.5 |
| Q6: Dressing/Hygiene (slope) | 0.0039 | 0.0223 | -1.9 |
| ALT (slope) | 0.0034 | 0.0166 | 0.1 |
| Q2: Salivation (slope) | 0.0032 | 0.0195 | 1.7 |
| Q9: Climbing Stairs (slope) | 0.003 | 0.0232 | -2 |
| AST (intercept) | 0.0028 | 0.0162 | 0.8 |
| FVC: Percent Normal (slope) | 0.0025 | 0.0225 | -3.4 |
| Race | 0.0021 | 0.0156 | -0.1 |
| ALT (intercept) | 0.0021 | 0.0182 | -0.7 |
| Bilirubin Total (slope) | 0.0019 | 0.0196 | -0.5 |
| Respiratory Rate (intercept) | 0.0017 | 0.0146 | 0.3 |
| Q2: Salivation (intercept) | 0.0013 | 0.0203 | -1.4 |
| Creatinine (intercept) | 0.0011 | 0.0152 | -0.7 |
| Age | 0.001 | 0.0185 | -2.1 |
| Q1: Speech (intercept) | 0.001 | 0.0224 | 2.2 |
| Potassium (slope) | 0.001 | 0.0136 | -0.9 |
| Onset Site: Bulbar | 0.001 | 0.0171 | -0.5 |
| Height | 0.0009 | 0.0169 | 0.8 |
| Weight (slope) | 0.0009 | 0.0174 | -1.7 |
| Sodium (intercept) | 0.0008 | 0.0188 | 1 |
| Bilirubin Total (intercept) | 0.0008 | 0.0183 | 0.8 |
| Sex | 0.0006 | 0.0169 | -0.4 |
| Q10: Respiratory (slope) | 0.0006 | 0.0153 | 0.8 |
| Q4: Handwriting (slope) | 0.0006 | 0.0206 | -0.7 |
| Weight (intercept) | 0.0006 | 0.0181 | -0.5 |
| Q5: Cutting (intercept) | 0.0002 | 0.0224 | -4.1 |
| Q3: Swallowing (slope) | -0.0003 | 0.0214 | -0.3 |
| Q10: Respiratory (intercept) | -0.0005 | 0.0189 | -0.4 |
| Albumin (slope) | -0.0006 | 0.0183 | 0.2 |
| Albumin (intercept) | -0.0006 | 0.0184 | -0.6 |
| Creatinine (slope) | -0.0007 | 0.0192 | 1 |
| Chloride (slope) | -0.0008 | 0.0201 | -0.9 |
| Used Riluzole | -0.0009 | 0.0196 | -0.6 |
| FVC: Percent Normal (intercept) | -0.0012 | 0.0177 | -0.3 |
| Blood Urea Nitrogen (intercept) | -0.0012 | 0.017 | -1.5 |
| Potassium (intercept) | -0.0012 | 0.015 | 0.6 |
| Q3: Swallowing (intercept) | -0.0015 | 0.016 | -0.5 |
| FVC: Subject Raw Liters (intercept) | -0.0016 | 0.0216 | -1.9 |
| Sodium (slope) | -0.0018 | 0.0187 | -0.2 |
| Chloride (intercept) | -0.0021 | 0.0188 | 0.2 |
| BloodUrea Nitrogen (slope) | -0.0022 | 0.02 | -1.2 |
| Respiratory Rate (slope) | -0.0027 | 0.0174 | -1.4 |
| Q7: Turning in Bed (intercept) | -0.0039 | 0.029 | -5.9 |
| Q4: Handwriting (intercept) | -0.0043 | 0.0209 | -4.2 |
| Q6: Dressing/Hygiene (intercept) | -0.005 | 0.0238 | -7.8 |
| Q8: Walking (intercept) | -0.0123 | 0.0226 | -1.7 |
| Q9: Climbing Stairs (intercept) | -0.0139 | 0.0232 | -2.8 |
